# REDUCING UNPLANNED HOSPITALIZATIONS OF NURSING HOME RESIDENTS: A PROCESS EVALUATION OF INTERCARE

**DOI:** 10.1093/geroni/igad104.2297

**Published:** 2023-12-21

**Authors:** Franziska Zuniga, Thekla Brunkert, Kornelia Basinska, Raphaëlle-Ashley Guerbaai, Christine Serdaly, Nathalie Wellens

**Affiliations:** University of Basel, Basel, Basel-Stadt, Switzerland; University of Basel, Basel, Basel-Stadt, Switzerland; University of Basel, Basel, Basel-Stadt, Switzerland; The Institute of Nursing Science, Basel, Basel-Stadt, Switzerland; Serdaly&Ankers snc, Conches (CH), Geneve, Switzerland; La Source School of Nursing, HES-SO University of Applied Sciences and Arts Western Switzerland, Lausanne, Vaud, Switzerland

## Abstract

The INTERCARE study provided evidence for a complex intervention to reduce unplanned hospitalizations in 11 Swiss nursing homes. The six intervention components were strengthening of interprofessional collaboration, introduction of a nurse with in-depth geriatric training (INTERCARE nurse), comprehensive geriatric assessment, advanced care planning, evidence-based tools, and data-driven quality improvement. This process evaluation aims to describe the intervention components’ use in practice and understand the mechanisms of change to optimize intervention components for scale-up. We used qualitative data from individual and focus group interviews and meetings notes from the research team’s conversations with nursing home care workers and leadership, INTERCARE nurses and physicians from five nursing homes participating in the INTERCARE study. Data was extracted into spread sheets before starting inductive coding. Findings were summarized and consolidated in a conceptual model. In connection with the four intervention elements interprofessional collaboration, INTERCARE nurse, advanced care planning and evidence-based tools, we identified four intervention mechanisms, grouped into 1) those changing reasoning of care workers e.g., following a more structured approach to do things and 2) those enabling existing resources e.g., availability of a responsible contact person. We also identified behavior changes in care workers, including faster reaction to changes in resident situations, more comprehensive assessment of resident situations and improved communication which contributed to reductions in hospitalizations. Understanding how interventions work in practice is crucial for informing potential adaptations to the intervention and the implementation strategies. Our findings can help optimize the intervention components for scale-up, ultimately reducing unplanned hospitalizations from nursing homes.

